# Low contribution of health extension workers in identification of persons with presumptive pulmonary tuberculosis in Ethiopian Somali Region pastoralists

**DOI:** 10.1186/s12913-017-2133-3

**Published:** 2017-03-11

**Authors:** Fentabil Getnet, Abdiwahab Hashi, Sahardid Mohamud, Hassen Mowlid, Eveline Klinkenberg

**Affiliations:** 1 College of Medical and Health Sciences, Jigjiga University, P.O. Box: 1020, Jigjiga, Ethiopia; 2 Ethiopian Somali Regional Health Bureau, Jigjiga, Ethiopia; 3 KNCV Tuberculosis Foundation, The Hague, The Netherlands; 4 Department of Global Health, Academic Medical Centre, University of Amsterdam, Amsterdam Institute for Global Health and Development, Amsterdam, The Netherlands

**Keywords:** Health extension worker, Identification, Presumptive, Tuberculosis, Referral, Pastoralist, Somali region, Ethiopia

## Abstract

**Background:**

To accelerate the expansion of primary healthcare coverage, the Ethiopian government started deploying specially trained community health workers named Health Extension Workers (HEWs) in 2003. HEWs work on sixteen health service packages; one being tuberculosis (TB) control and prevention. However, their contribution to TB care and prevention services among pastoralist communities has not been evaluated. Thus, this study has assessed their contribution in identification of persons with presumptive pulmonary TB in Ethiopian Somali Pastoralist Region.

**Method:**

A cross sectional study with mixed approach of quantitative and qualitative methods was applied. A randomly selected cross-sectional sample of 380 pulmonary TB cases from 20 health facilities was selected to obtain information on the role of HEWs in the identification of persons with presumptive TB, and their referral. Purposively selected HEWs were also interviewed individually to obtain in-depth information on their in-service training and experiences with referring TB cases. SPSS version20 was used to summarize the quantitative data and test statistical significance using chi-square test and logistic regression model. The qualitative data was analyzed under the principles of thematic analysis.

**Result:**

Overall, 20.3% [95% CI = 16.6–24.5] of pulmonary TB patients were referred by HEWs; while the majority were referred by healthcare workers (52.6%), family members (13.4%), neighbours/friends (2.4%) and self-referred (11.3%). Out of all, 66.1% and 53.4% had neither received community TB health education nor home visit from HEW respectively. Multivariate analysis indicated that provision of community health education [AOR = 14.0, 95% CI = 6.6–29.5], being model household [AOR = 21.2, 95% CI = 9.5–47.3], home visit from HEW [AOR = 2.8, 95% CI = 1.2–9.6] and rural residence [AOR = 3.0, 95% CI = 1.2–7.7] were significantly associated with referral by HEW. The qualitative findings supported that HEWs’ involvement in referral of persons with presumptive TB was limited. Communities’ low confidence in HEWs, inaccessibility of TB services at nearest health centers and lack of in-service trainings for HEWs were identified by the interviewee HEWs as underlying factors for their limited involvement.

**Conclusion:**

The contribution of health extension workers in identifying and referring presumptive TB cases is limited in Ethiopian Somali pastoralist region. Increased community health education and home visits by HEWs could contribute to increased identification and referral of persons with presumed TB. HEW should be properly trained on TB through in-service refreshment trainings and supported by routine supervision. Further expansion of TB diagnostic services would benefit to increasing case detection.

## Background

Pastoralism is a way of life whereby the livelihood of the people depends on raising livestock and living on its milk and meat [1]. Nearly 90% of the population in Ethiopian Somali region resides in rural areas, leading either a pastoralist or agro-pastoralist lifestyle. The majority of these pastoralists are nomadic and have a pattern of seasonal movement from place to place for cattle grazing. This nature of unstable settlement poses challenges in accessing healthcare services including tuberculosis (TB) [2]. For instance, limited access to TB control services was reported as the most important barrier in early care seeking for TB among pastoralist patients in this region, with the nomadic pastoralists being most affected [2–4]. In addition, routine consumption of raw milk and the poorly ventilated small dam-shaped housing structures where the pastoralist families live under crowded condition may also enhance TB transmission in this area.

Results of the first Ethiopian national TB prevalence study indicated that the prevalence of pulmonary TB among pastoralists was just above the national prevalence [316 (163–468) versus 277 (208–347) /100,000 respectively] but obviously with a much broader confidence interval [5]. However, the prevalence of TB in the region could be beyond this since up to 3, 000 TB cases have been diagnosed per year [6]. Case detection among these populations is considerably lower than the target, given their mobility and limited access to TB care and prevention services [7–9].

The Ethiopian Federal Ministry of Health has introduced an innovative approach called the Health Extension Program (HEP) in 2003 to address especially rural public health challenges. The overall aim of the program is to achieve equitable access to preventive essential health interventions. The community based health services focus on sustained preventive health actions and increased health awareness to the rural and poor communities. The program has 16 health service packages with TB prevention and control as one package. Under the program, Health Extension Workers (HEWs), working at health posts level, are the backbone of rural health service provision in agrarian and pastoralist regions. Health posts from where HEWs operate are constructed in each *kebelle.* The *kebelle* is the smallest administrative unit in Ethiopia consisting of at least 500 households, or the equivalent of 3500 to 4000 persons [10].

HEWs are recruited based on nationally concurred criteria that include residence in the *kebelle*, knowledge of the local language, 10^th^ grade complete, and willingness to go back to the *kebelle* and serve the community. They receive one year pre-service training at technical, vocational, and educational training institutions with a short practical training in health centers. After graduation, two HEWs are assigned to a health post in their respective *kebelle,* where they were selected, to provide HEP health services. The government pays modest monthly salary. HEWs are all female except in pastoralist areas where male workers dominate, given the cultural and environmental factors are not supportive for females [10]. The HEWs split their time for health post and community outreach activities to provide basic services on 16 packages categorized into health education, family health, hygiene and sanitation, and disease prevention and control [11].

Different factors affect the TB case detection rate. These can be divided in patient related factors like health seeking behaviour and health system related factors like proximity of services/access to health facilities, diagnostic algorithm and tools used, quality of diagnostic services, but also health care provider related factors like suspicion index of presumptive cases, level of training of the care provider [12–14]. HEP is aimed to enhance active TB case-finding by identifying undiagnosed cases in the community. HEWs have been given a significant role to actively identify persons with presumptive TB (*suspected but not confirmed TB cases*) during their routine house-to-house visits, community meetings and consultation times, and to refer them to health centers and hospitals for diagnosis and care [10, 15, 16].

Results of a randomized community trial in Southern Ethiopia showed that providing in-service training to HEWs on presumptive TB case identification, involving them in sputum collection and provision of directly observed treatment significantly improved case detection in the area [17]. The contribution of HEWs to pastoralists’ TB services accessing has not been evaluated for the Ethiopian Somali pastoralist region where the standard approach might work differently unlike the agrarian in southern Ethiopia, given specific cultural and geographic characteristics. Therefore, this study investigated the contribution of HEWs in the identification of presumptive pulmonary TB cases in the Somali pastoralist community as well as the experiences of the TB patients in accessing TB services through involvement of HEWs. The results can assist the region in optimizing the approach for increasing case detection among the pastoralist community.

## Methods

This study was conducted in Ethiopian Somali Regional State. The region has a total population projection of 5,307,002 that consists of 2,887,001 (54.4%) male and 2,420,001 (45.6%) female by 2014. More than 85% of the total population resides in rural areas where most lead pastoral way of life [18]. There were 8 public hospitals and 32 public health centres providing TB services in the region. They provide TB services under the National Tuberculosis Control Program of Ethiopia. The peak of health system management structure is Regional Health Bureau (RHB). The next level is Woreda health office which governs health centers and satellite health posts. Hospitals are directly amenable to regional health bureau. The structure lacks Zonal health department but each zone has one Zonal focal person who serves as regional health bureau delegate to each zone.

A study employing a mixed approach of both quantitative and qualitative methods was conducted from June 2014 to November 2014. A cross-sectional study design was applied to obtain information on the role of HEWs in presumptive TB case identification and referral. In addition, in-depth interviews with HEWs were done to obtain information on their in-service trainings and experiences with referring TB cases.

Since HEWs do not have a formal record keeping mechanism for presumptive cases they refer, we used all pulmonary TB (PTB) patients in the region as source population for the quantitative study. The approach was to assess from TB cases on treatment; what kind of interaction they had had with the HEW. The sample size was calculated as 384 TB patients using single population proportion formula in Epi Info7 assuming that 50% of TB cases (with a 95% confidence level and 5% precision) had been referred by HEWs.

Probability sampling was used to select the PTB patients. From all health facilities providing TB care services in the region, half of the health facilities (20) (4 hospitals and 16 health centres) were randomly selected. In each selected facility, the list of TB patients under treatment at the facility was collected prior to data collection. Using this list the number of TB patients to be sampled was allocated proportionally to the case load in each selected facility. Finally, the study participants were randomly selected by simple lottery method at each facility. All PTB patients above 15 years old were included although severely ill PTB patients who could not respond to questions were excluded and replaced by other patients.

A structured, interviewer administered and pre-tested questionnaire was used. The questions were developed by referring the HEP manual, the study in southern Ethiopian on HEWs [17] and the study on role of HEWs in maternal health services [19]. Questions were tend to collect data on socio-demographic variables, referral pathway, receipt of community TB health education, perception of patients towards HEWs service and model household graduation. Model household means a household that is graduated for all 16 packages of the HEP. HEWs organize training for household heads on the 16 packages of the HEP. After completion of the training, the trainees will be awarded as ‘model household.

The questionnaire was developed in English and translated to Somali language. Then the translated questionnaire was pre-tested on 30 PTB patients to evaluate whether the questions effectively capture the objectives, check understandability of questions and identify common errors like missing, confusing and leading questions. Data collectors were Bachelor of Science (BSc) holder health professionals who had previous experience on both quantitative and qualitative data collection. Training was given for all data collectors to assure quality and uniformity. The interviews were conducted at the respective health facilities when patients came to take their drugs in the morning.

HEWs were the source population for the qualitative study. Saturation sampling was performed. Initially, five HEWs were selected purposefully from different Woredas for the key informant interview. A semi-structured interview guide was used to conduct the in-depth interview and interviews were ended when information saturation was reached.

All quantitative data were entered into EpiData version 3.1 and exported to SPSS version 16 & 20 for analysis. Descriptive statistics were performed to calculate mean, frequency and proportion for both dependent and independent variables. Chi-square test and binary logistic regression methods were performed to investigate the association between presumptive TB cases referral and patient and HEW related parameters collected. The predictor variables tested were socio-demographic of patients (sex, age, educational status, income, residence); distance/time to travel from patients’ home to nearest health post; community TB health education by HEW; home visit from HEWs; model house hold graduation; and perception of patients towards HEWs’ services. All Variables yielding a *P*-value of ≤0.3 in the bivariate analysis were included in the multivariate analysis within their pre-structured conceptual frame. A *p*-value of 0.05 and below was considered as statistically significant. In-depth interviews were audio recorded and transcribed in to Somali language and translated into English. Matrix analysis in Microsoft Excel was used to summarize the qualitative information obtained from the key informant interviews based on the principles of thematic analysis.

## Results

### Socio-demographic characteristics

A total of 380 PTB patients participated in the study with a mean age of 32.8 years old and a standard deviation of 17.8 years. Two thirds were between 16 to 45 years of age. The male to female ratio was 1.7: 1. Two thirds were rural inhabitants, either pastoralist or agro-pastoralist. Nearly two thirds were married while nearly three quarters were illiterate (Table 1).

**Table 1:** Socio demographic characteristics of PTB patients in Ethiopian Somali region, June to November, 2014

Variable (*n* = 380)		Frequency (%)
Sex	Female	142 (37.4)
	Male	238 (62.6)
Age Group	16 – 30	215 (56.6)
	31 – 45	84 (22.1)
	45+	81 (21.3)
Residence	Rural	283 (74.5)
	Urban	97 (25.5)
Marital status	Single	107 (28.2)
	Married	245 (64.5)
	Divorced	10 (2.6)
	Widowed	17 (4.5)
	Separated	1 (0.3)
Educational status	No school/illiterate	276 (72.6)
	Read and write	29 (7.6)
	Primary	46 (12.1)
	Secondary	21 (5.5)
	College and above	8 (2.1)
Occupation	Pastoralist	181 (47.7)
	Agro-pastoralist	21 (5.5)
	Gov. employee	21 (5.5)
	House wife	18 (4.7)
	Daily laborer	41 (10.8)
	Merchant	30 (7.9)
	Others	68 (17.9)
Income (ETB)	Less than 500	125 (32.9)
	500 – 1000	68 (17.9)
	1000 – 1500	79 (20.8)
	Above 1500	108 (28.4)
Family Role	Husband	143 (37.6)
	Wife	108 (28.4)
	Child	121 (31.8)
	Relative	8 (2.1)
Distance from nearest HP	Less than 30 min	199 (52.4)
	30 – 60 min	64 (16.8)
	More than 1 h	117 (30.8)

N.B: ETB is Ethiopian Birr; 1ETB = 0.05 USD

### Pulmonary tuberculosis case referral by HEWs

Overall, 20.3% [95% CI = 16.6–24.5] of the PTB patients were referred by HEWs, while about half (52.6%) were referred by health care workers. Others were referred by family members (13.4%), neighbour/friends (2.4%) or self-referred (11.3%). Only 15.3% (*n* = 58) of PTB patients indicated to have consulted a HEW as first point of care when they had cough and other TB related symptoms. More patients had consulted health care workers (42.1%) and family members (34.7%). HEWs were not available in all *Kebelles* in the region as 14.5% of PTB patients indicated that there was no HEW in their respective *Kebelles*.

Of the 77 PTB patients referred by HEWs, 32.5% (*n* = 25) received a referral paper while the rest received verbal referral information. In terms of timing, 75.3% (*n* = 58) of PTB patients visited the health center or hospitals within 1 to 2 days after being referred by the HEW, 13% (*n* = 10) within 3 to 7 days and the remaining 11.7% (*n* =9) delayed more than a week to visit the hospital or health center. Reported reasons for delayed care seeking were lack of money (8.2%), symptom (s) not considered urgent enough to seek care (31.7%) and distance to health facility too far (60.1%).

### PTB Community Health Education by HEWs

From all the interviewed PTB patients, 76.3% (*n* = 290) had never received community health education from a HEW and 14.5% (*n* = 55) of them were not aware of HEWs’ availability in their *kebelle*. Also, 53.4% (*n* = 203) of the PTB patients indicated their home had never been visited by a HEW. Of the 177 patients whose homes were visited, about half (53.1%; *n* = 94) received TB health education at the time of the home visit. The TB health education provided covered information on disease causing agent, transmission pathway and signs and symptoms of TB. Furthermore, 47.5% (*n* = 84) of those whose home was visited were asked by the HEW about the presence of a family member with a cough of more than two weeks at some of the visits. A quarter 25.4% (*n* = 45) of patients were asked this during the last visit.

Of all 380 PTB patients interviewed, 119 (31.3%) indicated that they had ever obtained one or more TB services provided by HEWs such as health education, symptom inquiry and referral. Of all the PTB patients who ever received TB services from HEWs, 66.1% indicated that they trusted the information obtained from the HEW while 65.2% rated the obtained information as important. Among the 380 patients, 17.9% (*n* = 68) were from a graduated model household. Of the 68 graduated, 73.5% (*n* = 50) were provided with TB education during the model household training.

### Factors associated with PTB referral

In multivariate analysis, patients who received health education were nearly 14 times [*p* < 0.001] more likely to have been referred by a HEW than those who had not. A home visit by a HEW nearly tripled the chance of being referred by a HEW [*p* = 0.001] while those from the rural areas were nearly 3 times more likely to have been referred by a HEW [*p* = 0.02]. In addition, PTB patients from model households graduated by HEP had the highest odds of being referred by HEWs compared to their counterparts [*p* < 0.001] (Table 2). However, other predictor variables tested had no statistically significant association with referral by HEW [*p* > 0.05] (Table 3).

**Table 2:** Multivariable logistic regression model of explanatory variables of PTB referral by HEW in Ethiopian Somali Region, 2014

Variable (*n* = 380)		Referral by HEW		*p*-value	AOR (95% CI)
		No (%)	Yes (%)		
Residence	Urban *(reference)*	82 (84.5)	15 (15.5)	0.02	1
	Rural	221 (78.1)	62 (21.9)		3.0 (1.8–2.8)
Community HE	No *(reference)*	270 (93.1)	20 (6.9)	<0.001	1
	Yes	33 (36.7)	57 (63.3)		14.0 (6.6–29.5)
Home Visit from HEW	No *(reference)*	194 (95.6)	9 (4.4)	0.001	1
	Yes	109 (61.6)	68 (38.4)		2.8 (1.2–6.9)
Time to travel to HP on foot	>30 min *(reference)*	137 (75.7))	44 (24.3)	0.4	1
	≤30 min	166 (83.4	33 (16.6)		0.8 (0.4–1.5)
Model HH graduate	No *(reference)*	286 (91.7)	26 (8.3)	<0.001	1
	Yes	17 (25.0)	51 (75.0)		21.2 (9.5–47.3)

*HP* Health Post, *HE* Health Education, *HEW* Health Extension Worker, *AOR* Adjusted Odds Ratio, *PTB* Pulmonary Tuberculosis. 1 in AOR column also implies reference group

**Table 3:** Bivariate analysis of plausible explanatory variables of PTB referral by HEW in Ethiopian Somali Region, 2014

Variable (*n* = 380)		Referral by HEW		*p*-value	COR (95% CI)
		No (%)	Yes (%)		
Sex	Female *(reference)*	113 (79)	30 (21)	0.8	1
	Male	190 (80.2)	47 (19.8)		0.9 (0.6–1.6)
Age	16–30	174 (80.9)	41 (19.1)	0.8	0.8 (0.4–1.5)
	31–45	66 (78.6)	18 (21.4)		1.0 (0.5–2.0)
	46+ *(reference)*	63 (77.8)	18 (22.2)		1
Educational status	No school	220 (79.7)	56 (20.3)	0.8	1.1 (0.6–2.1)
	Read & write	22 (75.9)	7 (24.1)		1.4 (0.5–3.9)
	Formal education *(reference)*	61 (81.3)	14 (18.7)		1
Residence	Urban *(reference)*	82 (84.5)	15 (15.5)	0.2	1
	Rural	221 (78.1)	62 (21.9)		1.5 (0.8–2.8)
Community HE	No *(reference)*	270 (93.1)	20 (6.9)	0.001	1
	Yes	33 (36.7)	57 (63.3)		23.0 (12.5–43.5)
Family Income (ETB)	Less 1000 *(reference)*	155 (80.3)	38 (19.7)	0.77	1
	Above 1000	148 (79.1)	39 (20.9)		1.1 (0.7–1.8)
Home Visit from HEW	No *(reference)*	194 (95.6)	9 (4.4)	0.001	1
	Yes	109 (61.6)	68 (38.4)		13.4 (6.5–28.0)
Time to travel to HP on foot	>30 min *(reference)*	137 (75.7))	44 (24.3)	0.06	1
	≤30 min	166 (83.4	33 (16.6)		0.6 (0.4–1.0)
Perception of patients towards HEWs’ TB services^a^	Not important *(reference)*	2	2	0.5	1
	Important	40 (34.8)	75 (65.2)		1.9 (0.3–13.8)
Trust of TB info. Obtained from HEWs^a^	Didn’t trust *(reference)*	4	3	0.2	1
	Trust	38 (33.9)	74 (66.1)		2.5 (0.6–12.2)
Model HH graduate	No *(reference)*	286 (91.7)	26 (8.3)	0.001	1
	Yes	17 (25)	51 (75)		33 (16.7–65.1)

*HP* Health Post, *HE* Health Education, *HEW* Health Extension Worker, *COR* Crude Odds Ratio, *ETB* Ethiopian Birr, *PTB* Pulmonary Tuberculosis. 1ETB = 0.05 USD. 1 in COR column also implies reference group. The symbol ^a^indicates the analysis is among patients who have ever received TB services. No % values for denominators less than 10

### TB services provided by HEWs; perceptions and experiences

A total of five HEWs were interviewed in-depth to reach saturation of sampling. Health education was described as the main service they provided in terms of TB control and prevention. In addition, they perform referral of presumptive TB cases if encountered during home visits. Treatment follow up during continuation phase was also mentioned as a service provided. In addition, it was mentioned that as part of TB service provision, they also assessed the housing condition like windows and number of persons sleeping per room during home visits.

The health education provided covered cause and risk factors for TB, signs and symptoms of TB, transmission pathway, how to prevent transmission, information on TB treatment and adherence. There was no similarity in the time, place and frequency of health education sessions provided by HEWs; some used posters but others did not use information, education and communication materials; venue varied from home to village meeting sites to markets; the frequency varied from once per week to once per month while the time per session varied from 15 to 40 min.

Their experience on presumptive TB case referral was limited to a few cases according to their response. Reasons indicated for the limited numbers referred were unavailability of TB services at the nearest health centres where HEWs had a referral linkage and therefore they verbally instructed presumptive cases to go to health facilities in town to access TB services. Also they encountered a limited number of presumptive TB cases in their *kebelles*. One HEW mentioned lack of confidence in HEWs by the population as a reason affecting referrals. Because of lack of confidence by the population, presumptive cases did not seek advice from HEWs when they had symptoms. The interviewed HEWs indicated they would refer presumptive cases, i.e. persons with productive or dry cough, appetite loss, weight loss, fatigue, fever, chest pain and night sweating of more than two weeks if they encountered them. One HEW indicated that they started to conduct general home visits only two weeks prior to the study interview just after they had received refresher training.

Despite their limited experience, there were HEWs who claimed they refer up to 10 presumptive cases per month to their nearest health centre although most referred cases were diagnosed negative for TB. One HEW who never referred a presumptive TB case said *"Most of the time, children and adults complain of a cough which is not more than 3 days and they don’t have any other signs and symptoms of TB. So, I did not come across any presumed TB person in my career."*

The HEWs indicated that overall community members accept and comply with their advice. However, they sometimes face challenges from a few individuals who show resistance to accept their advice and lack of trust resulting in poor health seeking behaviour because of personal or cultural beliefs. For instance, one HEW explained his experience with one old woman as follows:

"She is an old woman and she usually doesn’t listen to people. She will tell you that she is not a child in need of advice. This is related to cultural issues. For instance, I told her to boil the milk before she consumes and she resisted because usually the nomads don’t boil milk and they prefer the raw milk. So instead of wasting time to this kind of people whose cultural issues are deep rooted, it’s better to talk to someone moderate in the family who can at least understand what I’m trying to tell him/her. In case of this old lady, I talked to her daughter and she in turn tried to make her understand. I try my best to change people’s perceptions and hopefully with Allah’s help things will change for better. For us, dealing with rural people is a hell on earth."

HEWs mentioned different misconceptions on TB causes and treatment perceived by the community. The main observed misconceptions with regards to the cause of TB are carrying of a heavy load, exposure to cold breeze and taking bath while sweating or coming out of the house into cold air while sweating. Community members believe these factors lead to body pain and chest pain and if these develop to cough, it leads to TB. One of the misconceptions on treatment is the wish of the people to take TB drugs even when they are diagnosed as TB negative. The other one is taking sheep fat as treatment although it was indicated that the practice is currently decreasing. Young people have a tendency of taking TB drugs to gain body weight and they request health professionals to give them TB drugs despite a negative diagnosis. Home remedies like honey and garlic, and egg yolk to a lesser extent are also commonly used for cough. The people also used amoxicillin for self-medication of respiratory syndromes according to the respondents.

### TB suspect referral systems

Most respondent HEWS had no formal referral papers to refer presumptive TB cases. It was indicated that verbal referral is more practical than paper. Also, some did not use a formal form but a blank paper to write the name and age of the presumptive TB case prior to referral. Some HEWs received feedback from health centres verbally through mobile phone but none ever received a formal written feedback about their referred presumptive TB cases.

### TB In-service training

Part of the HEWs had received in-service training just 1or 2 months prior to the interview. The contents of the training included causal pathway, transmission route, prevention of TB infection, signs and symptoms of TB, treatment follow up and monitoring of medication, process of referral that is how to link presumptive TB cases to the health center and TB/HIV co-infection including the screening of TB patients for Human Immuno-deficiency Virus (HIV). The trainings were organized by the RHB. All interviewed HEWs indicated that they received in-service training only once.

Some respondents acknowledged the support given by the Woreda Health office and RHB, and ranked it as good. Others responded the support is not sufficient. They stressed the importance of TB trainings and indicate that no Non-governmental organization was supporting their activities on TB service provision.

## Discussion

The purpose of this study was to investigate the contribution of HEWs in PTB suspect identification and referral and its associated factors in the case of Somali region pastoralist community where HEWs are the backbone of the health system. The results of the study indicate that the contribution of HEWs in presumptive PTB case identification and referral was limited as only one fifth of PTB patients on treatment were referred by a HEW. This is not in line with the government case finding strategy wherein HEWs have been given the responsibility to identify the majority of rural presumptive PTB cases as they work close to the community [20]. The majority of the PTB patients interviewed had been referred by other health care workers (52.6%).

This low referral of presumptive PTB cases by HEWs could be due to the lack of community health education by HEWs. Providing health education and home visits are key tasks of the HEWs [10], however, three quarters of interviewed PTB patients had never received health education from a HEWs and more than half (53.4%) of the patients had never been visited by a HEW in their home. The finding of this study revealed that those patients provided with TB health education by HEWs were nearly 15 times more likely to be referred by a HEW.

Low levels of in-service training of HEWs could be the underlying factor for the limited role they played as suggested by the qualitative findings. This is similar with the study done in Southern Ethiopia which showed that HEWs trained in provision of TB services, i.e. how to identify suspects, collect sputum, and provide directly observed treatment, increased case notification and detection rate by 52% compared to non-trained HEWs [17]. The other could be inadequate pre-service training. A review of available evidences has revealed the HEWs have been trained by the old curriculum which is expressed as a large curriculum with a short training period. This curriculum prescribes 70% practical training but 95% of the courses do not have practical training due to too few facilities to give practical lessons [21].

Lack of confidence in HEWs combined with poor health seeking behaviour linked to personal or cultural beliefs of the community affect the receptiveness of HEWs advice as shown by our qualitative findings.

On the other hand, HEWs are engaged in a wide range of disease prevention and health promotion activities in their respective *Kebelles* and therefore cannot devote all their time to TB care and prevention. For instance, a study done in northern Ethiopia showed that HEWs have contributed substantially to the improvement in women’s utilization of family planning, antenatal care and HIV testing, but their contribution to the improvement in health facility delivery, postnatal check-up and use of iodized salt was insignificant [19]. Moreover, it has been found HEWs have preferred some HEP services to others, mainly delivery as a preferred service in most regions of the country including Somali [22]. This might be indicative for the fact that HEWs do not devote equal time to all Health Extension Program packages.

Distance to health posts did not affect referral by HEWs to TB care centers for diagnosis and treatment services. This was contrary to expectation as an earlier study done in Somali region in 2007 on delay in diagnosis of TB concluded that the only observed risk factor for long patient delay of >120 days, was distance to health facility [2]. This might be due to the expansion of health facilities in the recent years up to *Kebelle* level [6].

A limitation of this study was that the data was collected from diagnosed PTB patients only. This introduces bias as not all presumptive TB cases referred by HEWs will arrive at the health facility and will be diagnosed as TB patients. Therefore, the observed figure of 20% of TB cases being referred might be an underestimate and the actual contribution of HEWs to the referral of presumptive cases might be larger. HEWs in the region do not systematically record presumptive PTB cases they refer while also health facilities did not systematically record by whom presumptive cases were referred and who were diagnosed not to have TB. The lack of a well-organized documented referral system makes it impossible to evaluate this. In addition, PTB patients might not recall if they were verbally referred by a HEW or they might not consider it as a referral which could also underestimate the rate of presumptive PTB case identification and referral by HEWs.

## Conclusion

Our findings indicated that there is a limited contribution of HEWs in presumptive PTB case identification and referral. More systematic/routine provision of community health education and household visits by HEWs could likely increase their contribution to TB case-finding. Traditional beliefs and perceptions about TB disease and lack of confidence in HEWs’ knowledge and skills still exist in the community according to the perception of the HEW. Moreover, provision of in-service training on TB to HEWs was limited. Hence, systematic provision of training to HEWs followed by supportive supervision could contribute significantly to finding the missing cases. Consequently, refreshment trainings on how to identify and refer presumptive cases, and provide directly observed treatment and follow up should be strengthened by the Woreda Health Offices and Regional Health Bureau. This helps HEWs to improve their knowledge and skills on TB services and to influence HEWs to put emphasis on tuberculosis. Further expansion of tuberculosis services would improve access to services for those being referred from the health post level.

A future prospective study could provide further insight in the role HEW play in referral of presumptive TB patients and the yield of TB diagnosis among the cases referred.

## Abbreviations

AOR: Adjusted odds ratio
CI: Confidence interval
COR: Crude odds ration
HEP: Health extension program
HEW: Health extension worker
HIV: Human immuno-deficiency virus
PTB: Pulmonary tuberculosis
RHB: Regional health bureau
TB: Tuberculosis

## References

[1] Montavon A, Jean-Richard V, Bechir M*.* Health of mobile pastoralists in the Sahel: Assessment of 15 years of research and development. Trop Med Int Health*.* 2013; doi:10.1111/tmi.12147. 10.1111/tmi.12147 23834073

[2] Gele AA, Bjune G, Abebe F (2009). Pastoralism and delay in diagnosis of TB in Ethiopia. BMC Public Health.

[3] Gele AA, Sagbakken M, Abebe F, Bjune GA (2010). Barriers to tuberculosis care: a qualitative study among Somali pastoralists in Ethiopia. BMC Res Notes.

[4] Gele AA, Bjune G. Armed conflicts have an impact on the spread of tuberculosis: the case of the Somali Regional State of Ethiopia. Confl Heal. 2010; 4(1). 10.1186/1752-1505-4-1 PMC2832778 20181042

[5] Kebede AH, Alebachew A, Tsegaye ZF, Lemma E, Abebe A, Agonafir M, Kebede AJ, Demissie D, Girmachew F, Yaregal Z, et al. The first population-based national tuberculosis prevalence survey in Ethiopia, 2010-2011. Int J Tuberc Lung Dis. 2014;18(6):635–339(5). http://dx.doi.org/10.5588/ijtld.13.0417. 10.5588/ijtld.13.0417 24903931

[6] ESRHB (2014). Regional health sector performance report. Edited by HIV/AIDS Ta.

[7] WHO (2014). Global tuberculosis report.

[8] WHO (2009). Global Tuberculosis Control. Geneva.

[9] FMOH (2014). Why TB? Evaluating the National TB Control Program: Challenges and ways forward. In: The 16th National Annual Review Meeting Group Discussion: 2014.

[10] FMOH (2007). Health extension program profile in Ethiopia.

[11] UNICEF (2014). The Extension Program: Survival and Health.

[12] Storla DG, Yimer S, Bjune GA (2008). A systematic review of delay in the diagnosis and treatment of tuberculosis. BMC Public Health.

[13] Yimer S, Bjune G, Alene G (2005). Diagnostic and treatment delay among pulmonary tuberculosis patients in Ethiopia: a cross sectional study. BMC Infect Dis.

[14] Yimer S, Holm-Hansen C, Yimaldu T, Bjune G (2009). Evaluating an active case-finding strategy to identify smear-positive tuberculosis in rural Ethiopia. Int J Tuberc Lung Dis.

[15] FMOH (2012). Guidelines for Clinical and Programatic Manageent of TB, Leprosy and TB/HIV in Ethiopia. Edited by TB L, TB/HIV.

[16] Yassin MA, Datiko DG, Tulloch O, Markos P, Aschalew M, Shargie EB, Dangisso MH, Komatsu R, Sahu S, Blok L (2013). Innovative community-based approaches doubled tuberculosis case notification and improve treatment outcome in Southern Ethiopia. PLoS One.

[17] Datiko DG, Lindtjorn B (2009). Health extension workers improve tuberculosis case detection and treatment success in southern Ethiopia: a community randomized trial. PLoS One.

[18] CSA (2013). Population Projection of Ethiopia for All Regions: At Wereda Level from 2014 – 2017.

[19] Medhanyie A, Spigt M, Kifle Y, Schaay N, Sanders D, Blanco R, GeertJan D, Berhane Y (2012). The role of health extension workers in improving utilization of maternal health services in rural areas in Ethiopia: a cross sectional study. BMC Health Serv Res.

[20] Workie NW, Ramana G (2013). The health extension program in Ethiopia.

[21] Banteyerga H (2011). Ethiopia's health extension program: improving health through community involvement. MEDICC Rev.

[22] Center for National Health Development in Ethiopia CU (2011). Ethiopia Health Extension Program Evaluation Study, 2007-2010, Volume-II. Health post and HEWs performance Survey.

